# The cytosol activity of thymidine phosphorylase in endometrial cancer

**DOI:** 10.1186/1756-9966-27-64

**Published:** 2008-11-05

**Authors:** Elżbieta Miszczak-Zaborska, Robert Kubiak, Andrzej Bieńkiewicz, Jacek Bartkowiak

**Affiliations:** 1 Department of Medical Biochemistry, Medical University of Lodz, 6/8 Mazowiecka Street, 92-215 Lodz, Poland; 2 Department of Tumour Pathology and Oncology, Medical University of Lodz, Paderewskiego Street 4, Lodz, Poland; 3 Department of Gynecology and Gynecological Oncology, Medical University of Lodz, Paderewskiego Street 4, Lodz, Poland

## Abstract

**Background:**

Thymidine phosphorylase (TP) is identical with platelet-derived endothelial cell growth factor (PD-ECGF) which promotes angiogenesis. The aim of this study was to evaluate the cytosol activity of TP in tumor samples from patients with endometrial cancer.

**Methods:**

The activity of TP was measured by the spectrophotometric method in the cytosol of endometrial tumor samples from 43 patients. Moreover, the expression of platelet-derived endothelial cell growth factor/thymidine phosphorylase (PD-ECGF/TP) protein and microvessel density (MD) were examined in the same endometrial tumor samples by immunohistochemical staining. Normal endometrium from 16 women, treated surgically due to nononcological reasons served as a control.

A relationship between the cytosol TP activity, PD-ECGF/TP protein expression, MD and clinicopathologic features was investigated.

**Results:**

A significantly higher the cytosol TP activity, PD-ECGF/TP protein expression and MD was stated in malignant tumor samples when compared to the control (samples of normal endometrium). A positive statistically significant correlation between the cytosol enzyme activity and PD-ECGF/TP protein expression and MD was found, but weaker from the remaining ones between PD-ECGF/TP protein expression and MD was observed.

Besides no correlation between the cytosol TP activity, PD-ECGF/TP protein expression as well as MD and grading or histopatological type of endometrial cancer was stated.

**Conclusion:**

The cytosol TP activity in endometrial cancer is significantly higher than in normal endometrium, with no relation as to the stage and grade of tumors, but correlates with the PD-ECGF/TP protein expression and MD may therefore be associated with favorable prognosis in patients treated with chemo- or radiotherapy after surgery.

## Background

Thymidine phosphorylase (TP: EC 2.4.2.4.) catalyses the reversible phosphorolysis of thymidine to thymine and 2-deoxyribose-1-phosphate [1]. TP is identical with platelet-derived endothelial cell growth factor (PD-ECGF) which promotes angiogenesis [1-6]. TP activity, PD-ECGF/TP protein and PD-ECGF/TP gene expression are significantly increased in tumor tissues as compared to the adjacent non-neoplastic tissues in a variety of human carcinomas [7-9]. In certain tumors a high TP activity, as well as a high of PD-ECGF/TP protein and PD-ECGF/TP gene expression are associated with unfavorable prognosis [10-12]. On the other hand, the high TP activity and the PD-ECGF/TP protein expression are suggested to be useful prognostic factors for tumors treated with chemotherapy or radiotherapy [13-15].

Endometrial carcinoma is one of the most common malignancies of the female genital tract. The increase of cytosol TP activity and a higher PD-ECGF/TP protein and PD-ECGF/TP gene expression as compared to the adjacent non-neoplastic tissues were reported in other gynecologic malignancies [9,16-18]. The data on PD-ECGF/TP protein expression and its correlation with microvessel density (MD) in endometrial cancer are controversial [19-21]. The cytosol TP activity and its correlation with MD in endometrial cancer has not been carried out so far.

The aim of the study was also to evaluate the influence of the cytosol TP activity and PD-ECGF/TP protein expression on the intensity of angiogenesis in malignant tumors enabling prognosis and choice of the proper therapy for patients with endometrial cancer after surgery.

## Methods

### Patients

This study included 43 patients with histologically confirmed endometrial tumors who underwent surgery at the Department of Gynecologic Oncology, Medical University of Łódź in Poland during the period from 2002 to 2005. No patient had received any therapy before surgery. All patients with adenocarcinoma were postmenopausal, aged between 50 to 88 years (mean age = 60). 16 women aged 32 – 44 years with normal endometrium, treated surgically (7 women were in the luteal and 9 were in follicular phase of the menstrual cycle) due to nononcological reasons served as a control.

The patients were staged according to the criteria recommended by the International Federation of Gynecology and Obstetrics (FIGO) [22]: stage I, n = 29; stage II, n = 7: stage III, n = 7. The tumors were histologically classified into three grades [23]: G-1, n = 18; G-2, n = 19; G-3, n = 6.

### Histology and immunohistochemistry

For histology hematoxillin and eosin staining were performed. For the immunohistochemistry staining, routinely processed, formalin-fixed, paraffin embedded, tissue blocks were cut on silanized slides.

Microvessel assessment was performed using a mouse anti-human CD31 (Dako) antibody in dilution 1:40 with streptavidin-biotin technique following prolonged (13 min) enzymatic digestion with trypsin. Microvessel counting, performed according to the Weidner's method [24] was initiated in the areas of most intensive vascularization (hot-spots) identified by scanning of the specimens at low power magnification. Counting was continued in ten consecutive high power fields (400×).

Immunohistochemical staining for PD-ECGF/TP protein was performed using the anti-TP mouse monoclonal antibody NCL-PDEGF clone P-GF.44C (NovoCastra) in dilution 1:30 [25]. The extent of immunohistochemical expressions of PD-ECGF/TP protein of specimens was classified into 0, 1, 2, 3 grades. Specimens graded as 1, 2, 3 were regarded as positively stained and those graded as 0 as negatively stained. We assumed cases as positive in which we observed more then 5% positively stained cells under high power magnification. However, in a part of cases we observed a significant heterogeneity of expression in tumor (two cellular populations mixed with/without expression). Then we accepted only these tumors as positive in which the percentage of positive fields amounted to over 5% of the whole tumor.

### Preparation of the cytosol fraction

Tissue samples (approximately 2 g) were dissected from tumors immediately following surgery and frozen at low temperature (-80°C) until required. Nonneoplastic endometrial samples were frozen, too. Both kinds of specimens were studied in the same conditions with the aid of the following methods: Tissue samples were homogenized in Ultra/turrax T 25 in 4 vol. of ice cold buffer (1 mM EDTA, 0.02% mercaptoethanol, 2 mM phenylmethanesulfonyl fluoride (PMSF), 10 mM tris (Hydroxymethyl) aminomethane – maleate, pH 6,5), with 10% glycerol and then centrifuged at 100 000 × g for 1 h, to obtain the cytosol fraction. It was then pooled and directly taken for analysis.

### Enzyme assay

TP activity was assessed by the spectrophotometric method described by Yoshimura et al. [7] in our own modification [16] using the transformation of thymine (T) from thymidine (dThd) in the presence of arsenate. The incubation mixture of 0.5 ml final volume contained 0.1 M tris (hydroxymetyl) aminomethane-arsenate buffer (pH 6.5), 10 mM dThd and appropriate volume of the cytosol fraction. After a 1 h incubation at 37°C, the reaction was stopped by adding 0.5 ml 1 N NaOH and the thymine formed was measured with absorbance at 300 nm. The protein content was determined according to the method described by Bradford [26]. The activity of TP was determined in micromoles of thymine released during 1 h per milligram of protein.

### Statistical analysis

The p < 0.05 was taken as the level of statistical significance. Univariate and multiple linear regression was used to analyse the data. All stastistical analyses were performed using a statistical software STATA 6.

## Results

### Activity of TP

**Figure 1:**
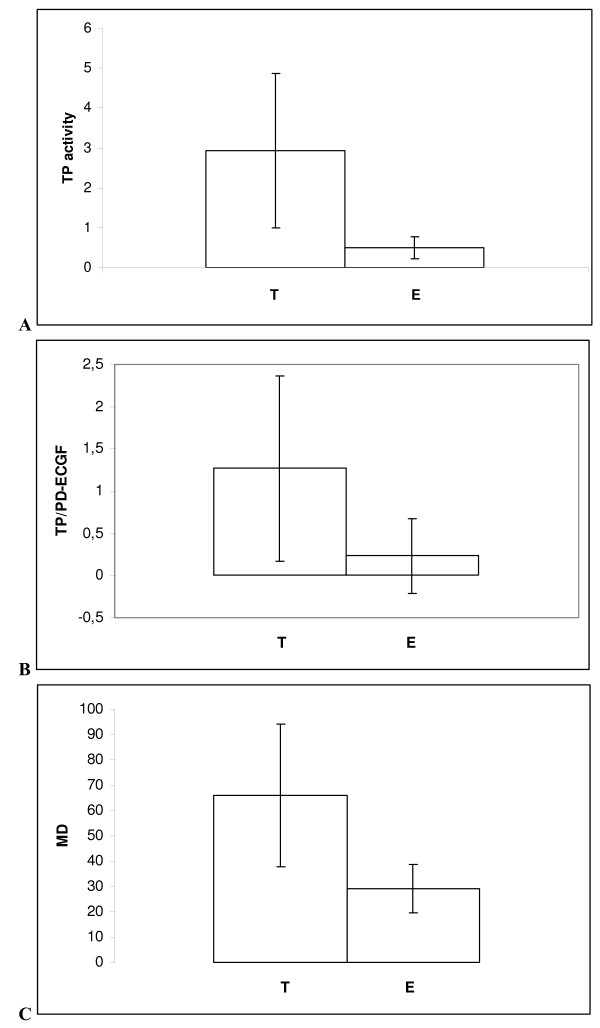
**The cytosol TP activity, PD-ECGF/TP – protein expression and microvessel density in tumor (T) and normal endometrium (E)**. Results are expressed as mean ± SD (standard deviation), p < 0.001. A.) TP activity – The cytosol TP activity measured in micromoles of T × mg protein^-1 ^× h^-1 ^as described in Materials and Methods. Tumor samples were from 43 endometrial cancer patients and normal endometrium from 16 women treated surgically due to nononcological reasons. B.) TP/PDECGF – PD-ECGF/TP – protein expression; The extent of immunohistochemical expressions was classified into 0, 1, 2, 3 grades as described in Materials and Methods. C.) MD – Microvessel density. Microvessel counting, performed according to the Weidner's method [24] measured as described in Materials and Methods.

TP activity was assessed by the spectrophotometric method described by Yoshimura et al. [7] in our own modification [16]. A significantly higher TP activity was stated in the cytosol of tumor samples from 43 endometrial cancer patients when compared to the control (normal endometrium from 16 women). The mean activity of TP measured in micromoles of T × mg protein^-1 ^× h^-1 ^in the cytosol from tumor samples it was 2.92 ± 1.96 (0.71 – 9.14) and from normal endometrial samples was 0.50 ± 0.27 (0.11 – 1.07) with p < 0.001 (Figure 1A).

### Expression of TP/PD-ECGF protein

**Figure 2:**
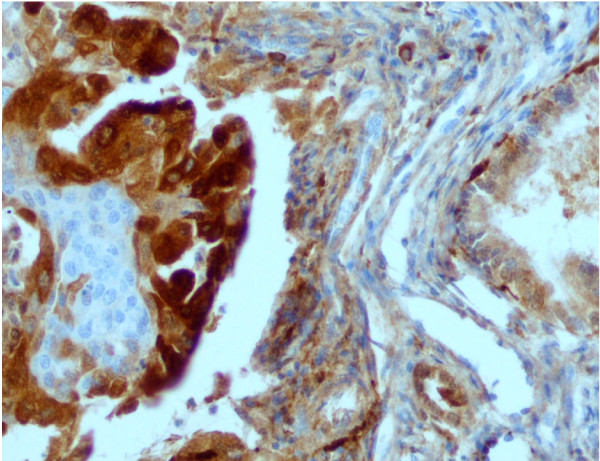
**Immunohistochemical staining for PDECGF/TP protein expression in endometrial cancer**. Immunohistochemical staining for PD-ECGF/TP protein was performed using the anti-TP mouse monoclonal antibody NCL-PDEGF clone P-GF.44C (NovoCastra) The cytoplasmic and only occasional nuclear staining of cancer cells is seen (note negative squamous metaplastic cells in the center). On the right normal endometrial cells. (200×).

**Figure 3:**
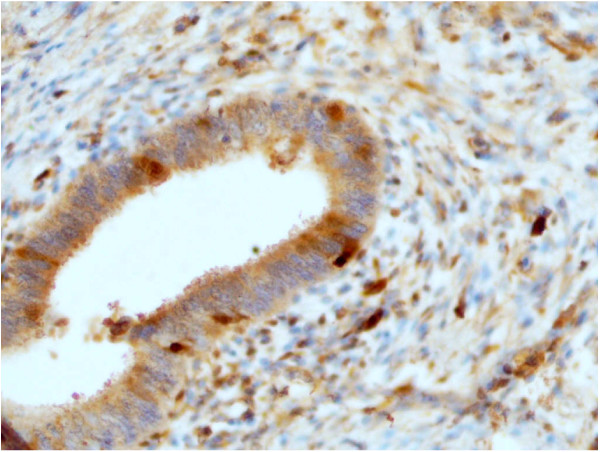
**Immunohistochemical staining for PDECGF/TP protein in normal endometrium**. Immunohistochemical staining for PD-ECGF/TP protein was performed using the anti-TP mouse monoclonal antibody NCL-PDEGF clone P-GF.44C (NovoCastra) (200×).

Microscopic features of specimens expressing PD-ECGF/TP protein are shown in figure 2 and 3. The extent of immunohistochemical expressions of PD-ECGF/TP protein of the specimens was classified into 0, 1, 2, 3 grades. Staining in endometrial tumor cells was mainly found in cytoplasm, sometimes in endothelial nuclei and part of stroma, possibly macrophages, granulocytes and fibroblasts (Figure 2.). From among the mentioned cells we only assessed epithelial ones. A significantly higher mean expression of PD-ECGF/TP protein was also stated in tumor samples from the endometrial cancer patients when compared to the control – samples from patients with normal endometrium (Figure 3): 1.24 ± 1.11 (0.00 – 3.00) and 0.23 ± 0.43 (0.00 – 1.00), respectively, with p = 0.001 (Figure 1B), measured as described in Methods.

### Microvessel density

**Figure 4:**
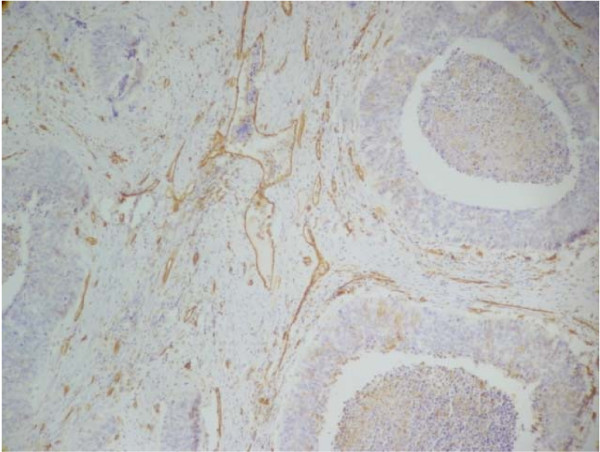
**Immunohistochemical staining for microvessel density (MD) in endometrial cancer**. Microvessel assessment was performed using a mouse anti-human CD31 (Dako) antibody (100×).

Microvessel counting, according to the Weidner's method [24] was initiated in the areas of most intensive vascularization (hot-spots) identified by scanning of the specimens at low power magnification (100×, Figure 4). Counting was continued in ten consecutive high power fields (400×). Control (normal endometrium) was the region with lower MD. MD in malignant tumors had a significantly higher value of microvessels: 67.18 ± 27.14 (24.00 – 117.3), in comparison with normal endometrium, where it attained the value 29.02 ± 9.64 (18.00 – 49.40) with p < 0.000001 (Figure 1C).

### Correlations

**Figure 5:**
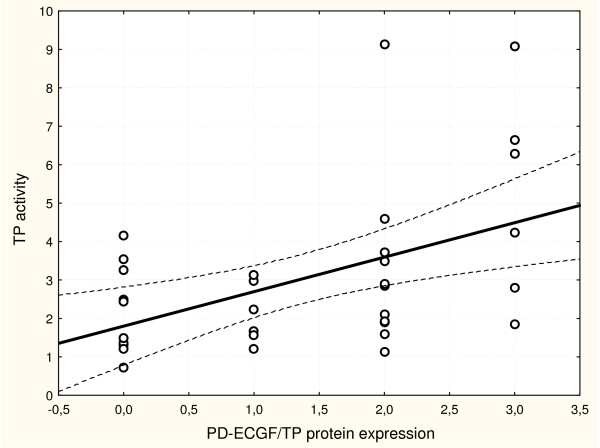
**The correlation between the cytosol TP activity and expression of PD-ECGF/TP protein in endometrial cancer**. p = 0.0035, R = 0.4858, statistical analysis according to Spearman (measured as described in Materials and Methods).

**Figure 6:**
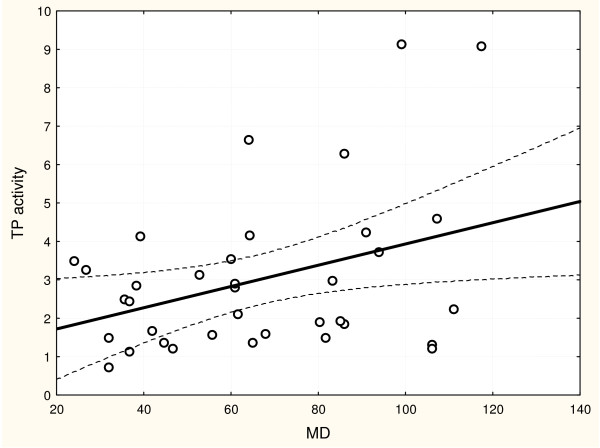
**The correlation between the cytosol TP activity and microvessel density (MD) in endometrial cancer**. p = 0.0286, R = 0.3649, statistical analysis according to Spearman (measured as described in Materials and Methods).

A correlation between the cytosol TP activity and PD-ECGF/TP protein expression and MD in malignant tumor samples of patients with endometrial cancers has been studied. A statistically significant positive correlation between the cytosol TP activity and PD-ECGF/TP protein expression (p = 0.0035, R = 0.4858; Figure 5.) was stated. A correlation between the cytosol TP activity and MD was also statistically significant (p = 0.0286, R = 0.3649; Figure 6), but weaker correlation between PD-ECGF/TP protein expression and MD (p = 0.0429, R = 0.3492 according to Spearman) was observed.

The consecutive statistical analysis was meant to find a correlation between the activity of the studied enzyme and clinical stages of endometrial cancer (assessed acc. to FIGO). As it follows from the presented data (Table 1) the cytosol TP activity is lower in more advanced neoplastic lesions (FIGO II and FIGO III versus FIGO I), however, no statistically significant dependence, has been found between the cytosol TP activity and clinical stages (p = 0.3471, R = -0.1487 – according to Spearman – Table 2). Similar observation and lack of correlation was stated for the expression of PD-ECGF/TP protein and clinical stages (p = 0.3323, R = 0.1594) and for MD and clinical stages of endometrial cancer, (p = 0.3057, R = -0.1639 – Table 1 and 2).

**Table 1:** Comparison of the cytosol TP activity, PD-ECGF/TP protein expression and microvessel density with clinicopathological characteristics in endometrial cancer.

	**TP activity**		**PD-ECGF/TP**		**MD**	
	
**Variables**	**Mean**	**SD**	**Mean**	**SD**	**Mean**	**SD**
**Grading**						

G-1	2.3572	1.9102	1.3125	0.3464	68.7058	25.4
G-2	3.4532	2.1369	1.3529	1.2217	65.47	28.04
G-3	2.6980	1.0889	0.5714	0.9759	64.6	32.29

**Stage**						

FIGO I	3.0938	2.0959	1.0769	1.0926	70.1892	27.35
FIGO II, FIGO III	2.6423	1.7258	1.4615	1.1982	60.3769	27.75

- TP activity in micromoles of T × mg protein^-1 ^× h^-1^, PD-ECGF/TP protein expression and microvessel density (MD) – measured as described in Materials and Methods

- clinical stages (FIGO I, FIGO II, FIGO III); grading (G-1, G-2, G-3)

**Table 2:** Relations between the cytosol TP activity, PD-ECGF/TP protein expression, microvessel density (MD) and clinicopathological characteristics in endometrial cancer.

		***Relations***	p	R
1	a	FIGO I/FIGO II, FIGO III vs TP activity	0.3472	-0.1487
	b	G 1, G 2, G 3 vs TP activity	0.0789	0.2741

2	a	FIGO I/FIGO II, FIGO III vs PDECGF/TP	0.3323	0.1594
	b	G 1, G 2, G 3 vs PDECGF/TP	0.1792	-0.2166

3	a	FIGO I/FIGO II, FIGO III vs MD	0.3057	-0.1639
	b	G 1, G 2, G 3 vs MD	0.5923	-0.0851

a) clinical stages (FIGO I/FIGO II, FIGO III); b) grading (G-1, G-2, G-3) (statistical analysis according to Spearman).

The cytosol TP activity, PD-ECGF/TP protein expression and MD were studied according to the histopathological degree of differentiation (Table 1). No statistically significant relation between the TP activity and grading (p = 0.0789, R = 0.2741), between the expression of PD-ECGF/TP protein (p = 0.1792, R = -0.2166) and between MD and grading of endometrial tumors (p = 0.5922, R = -0.0851) (Table 2) has been found.

No statistically significant relation between TP activity, PD-ECGF/TP protein expression, MD and age and tumor diameters of patients was concluded, either (data not published).

## Discussion

Thymidine phosphorylase (TP) is identical with platelet-derived endothelial cell growth factor (PD-ECGF) which promotes angiogenesis [2-6]. The aim of our study was to evaluate the cytosol activity of TP together with its protein level (PD-ECGF/TP protein expression) in relation to the intensity of angiogenesis in endometrial cancer.

Fourty three patients with endometrial malignant tumor were included in the study. The control was the normal endometrium obtained from sixteen patients. The enzyme activity was measured in the cytosol from tumor and normal tissues using the spectrophotometric method and expressed in micromoles of thymine released during 1 h per milligram of protein. A significantly higher mean cytosol TP activity was stated in these malignant tumors as compared to the control. We obtained similar observations for cytosol TP activity from ovarian [17] and breast carcinoma [16]. In benign and mixed uterine tumors [9] we also found a significantly higher mean TP activity (in the cytoplasmatic soluble fraction obtained at 50 000 × g and in partially purified fraction after DEAE-Sepharose) from only those women who were in the follicular phase of the cycle during surgery as compared to the control (myometrium), although the obtained TP activity values did not differ in the normal myometrium from women who were in luteal and in follicular phase of the menstrual cycle during surgery [9]. In our present data no statistically significant dependence has been found between the cytosol TP activities in normal endometrium in both phases of the menstrual cycle, either (data not published).

Our results are in agreement with these obtained by Takebayashi et al. [8] for several human solid tumors. TP activity was measured in tissue homogenate using the same spectrophotometric method as ours and expressed in micromoles of thymine released during 1 h per milligram of protein and was significantly higher in carcinomas of oesophagus, stomach, colorectum, pancreas and lung than in the adjacent non-neoplastic tissue. On the contrary, there was no significant difference between the TP activity in carcinoma of liver and thyroid and than in the control materials [8]. The high TP activity in tumors was associated with unfavorable prognosis [10-12], but there were published data that in patients treated with chemotherapy this high TP activity might contribute to better prognosis [13,14].

The enzyme was measured using the spectrophotometric method and expressed in nanomoles of 5-fluorouracil (5-FU) released during 1 minute per mg protein in the cytosol from cervical cancer (n = 20), leiomyoma (n = 23), ovarian cancer (n = 46), ovarian endometriosis (n = 21) benign epithelial ovarian tumor (n = 27) and also from endometrial cancer tissues (n = 26) [27]. This study indicated that he mean activity in endometrial cancer tissues was significantly higher (64,5 nanomoles of 5-FU × mg protein^-1 ^× minute^-1^) than in the corresponding normal tissues (27,5 nanomoles of 5-FU × mg protein^-1 ^× minute^-1^[27].

We have also measured the expression of PD-ECGF/TP protein in the endometrial cancer and normal endometrium using an immunohistochemical technique with the P-GF-44C monoclonal antibody. In our study the staining was mainly found in cytoplasm, sometimes endothelial nuclei and part of stroma. Our observations are similar with these obtained by Fujiwaki et al. for endometrial cancer [28]. In our study the expression of PD-ECGF/TP protein was statistically higher in endometrial cancer as compared to the control. No expression was detected in coexisting normal endometrial stroma or stroma of endometriosis either [29]. Immunohistochemical staining with a monoclonal antibody against TP was also used in the study of the cancerous oesophagus, stomach, colon, bladder, pancreas and lung, and the proportion of TP-postive tumors was also significantly higher than that of the TP-positive adjacent normal tissues [8]. However our results remain in controversy with those obtained by Sivridis et al. [30] who found a poor expression of TP in endometrial carcinoma. They reported, that immunopositiveness for PD-ECGF/TP was increased in a small proportion of endometrial carcinomas (approximately 5%) and up-regulation occurred predominantly at the invading tumor front which is probably induced by cytokines of histocystic and lymphocytic origin [30].

Takebayashi et al. [8] assayed the expression of PD-ECGF/TP protein and the activity of TP in the same human solid tumor specimens and found a correlation between them [8]. Little is known of TP activity in endometrial cancers except for the works of Kamoi et al. [31] (article and abstract in Japanase) and Suzuki [27]. Thus we have studied spectrophotometrically the cytosol TP activity in tumor samples and tested if it is correlated with immunohistochemically studied expression of PD-ECGF/TP protein in the same cases of well-differentiated endometrial carcinomas. Our study revealed that the expression of PD-ECGF/TP protein was correlated with the cytosol activity of TP in the same endometrial cancer, and this correlation was statistically significant.

In endometrial cancer a high microvessel count is an independent prognostic factor [32-34] as it has relation to metastases [35]. However, Seki et al. [36] reported, that MD was not associated with lymph node metastasis or surgical stage and therefore angiogenesis does not necessarily contribute to metastasis in endometrial cancer [36]. Abulafia et al. [37] reported that angiogenesis was increased in complex endometrial hyperplasia as compared to controls of simple hyperplasia. The angiogenic capability of complex hyperplasia was comparable to the FIGO stage Ia endometrial cancer. An increased angiogenesis was found in cases of invasive (stages Ib and Ic) endometrial cancer as compared to complex hyperplasia or stage Ia endometrial cancer [37]. Fujiwaki at al. [38] found that the immunohistochemical expression of TP was associated with the increase of MD as an index of angiogenesis in endometrial cancer, which suggested that TP might play an important role in angiogenesis [38]. In our study MD attained a significantly higher value in endometrial cancer in comparison with normal endometrium. Our data confirmed the correlation between MD and the cytosol TP activity and revealed the weaker of such a correlation between MD and PD-ECGF/TP protein expression in endometrial cancer, indicating that the active enzyme but not its protein influences in some way the vessel development i.e. that variety of factors may influence the activation of enzymatic protein. A high PD-ECGF/TP protein expression accompanying the high cytosol TP activity may concern also the cases of its localization in stroma. Also Sakamoto et al. [29] showed that immunohistochemical PD-ECGF/TP expression was predominantly observed in tumor stroma, and that TP-positive cells in the stroma are probably macrophages and plasma cells [29]. Seki et al. [36] found, that PD-ECGF production within cancer cells was not so strong as in the stroma cells and there was no correlation between the expression of PD-ECGF in cancer cells and any clinicopathological variables including microvessel count [36]. Another study failed to find a relationship between PD-ECGF expression and angiogenesis either [39]. In the study of Sivridis et al. [40] the expression of PD-ECGF in tumor cells did not effectively contribute to the angiogenic process and was deprived of any practical significance since no relationship was found between this expression and the histopathological factor examined, but was associated with aggressive histological features endometrial carcinomas [40]. In our study in endometrial cancer revealed no significant correlation between the cytosol TP activity, PD-ECGF/TP protein expression, MD and histopathological factors. Our results were in agreement with these obtained by Suzuki at al. [27]. There were no significant differences in the cytosol enzyme activity according to tumor stage classification. When assessed according to histopathologic degree of differentiation, the enzyme activity appeared to be higher in pathologic grade G – 3 than in grade G – 1 and G – 2, but this difference did not show significance [27]. We found that TP activity in tumor was higher in grade G – 2 than that obtained in G – 1 and G – 3 and this difference did not show significance either. The same concerns PD-ECGF protein expression. Our results are in conformity with these obtained by Mazurek et al. [41] who found no statistically significant correlation between TP immunohistochemical expression and histopatological grade and FIGO stage, but particular FIGO stages showed a significant trend of increasing TP endometrial cancer overexpression [41]. We also found that PD-ECGF/TP protein expression was higher in FIGO stage II and III vs I, but there were no significant differences, either.

The mechanism of endometrial angiogenesis involves stimulation by ovarian steroids of production of angiogenic regulators by endometrial epithelium and stroma which then act on the endothelium [42]. Endothelial cells are the principal actors in angiogenesis, which after effecting the proteolytic degradation of the basemant membranes, migrate through the membrane to form sprouts [43]. 2-deoxy-D-ribose generated by PD-ECGF/TP catalyzing phosphorolysis of thymidine, stimulates endothelial cell migration [1,44] via a mechanistic pathway similar to that described for glucose that attracts endothelial cells along its concentration gradient [45]. However, Brown et al. suppose, that 2-deoxy-D-ribose induces cellular oxidative stress, which generates oxygen radicals during early stages of protein glycation and promotes secretion of angiogenic factors [46,47]. Moreover, it was suggested that, beside 2-deoxy-D-ribose, thymine might also exhibit angiogenic properties stimulating the effects of other growth factor [48]. The extent to which TP is implicated in neoangiogenesis is far from being fully elucidated [49]. Although the mechanism of TP induction is not yet completely clear, but TNF, IL10 and other cytokines have been clearly shown to induce its expression. The various complex interactions of TP give it an essential role in cellular functioning and, hence, it is an ideal target in cancer therapy [50].

## Conclusion

Our results indicate, that the cytosol TP activity together with PD-ECGF/TP protein expression is higher in endometrial cancer when compared to normal endometrium and is correlated with angiogenesis. It suggests, that appropriately modulated TP activity may be an interesting target for a novel cancer drug in endometrial cancer. The study of TP activity together with PD-ECGF/TP expression and its correlation with angiogenesis may contribute to a proper choice of therapy for patients with endometrial cancer.

## Competing interests

The authors declare that they have no competing interests.

## Authors' contributions

EMZ conceived the study, performed the biochemical experiments and drafted the manuscript, RK performed the immunohistochemical experiments, helped to write the manuscript, AB collected tissue specimen, clinical records. JB conceived the idea, provided helpful comments. All authors read and approved the final manuscript.
